# Diacutaneous Fibrolysis Intervention in Patients with Mild to Moderate Carpal Tunnel Syndrome May Avoid Severe Cases in Elderly: A Randomized Controlled Trial

**DOI:** 10.3390/ijerph191710983

**Published:** 2022-09-02

**Authors:** Sandra Jiménez-del-Barrio, Luis Ceballos-Laita, Elena Bueno-Gracia, Sonia Rodríguez-Marco, Santos Caudevilla-Polo, Elena Estébanez-de-Miguel

**Affiliations:** 1 Clinical Research in Health Sciences Group, Department of Surgery, Ophthalmology, Otorhinolaryngology, and Physiotherapy, University of Valladolid, 47002 Valladolid, Spain; 2 Department of Physiatrist and Nursery, Faculty of Health Sciences, University of Zaragoza, 50009 Zaragoza, Spain

**Keywords:** Carpal Tunnel Syndrome, ultrasonography, physical therapy modalities, Diacutaneous Fibrolysis

## Abstract

Background: Carpal Tunnel Syndrome (CTS) mainly affects adults of working age. The prevalence of severe cases is higher in elderly patients (>65 years old). Clinical guidelines recommend conservative treatment as the best option in the initial stages of CTS to avoid severe cases. Diacutaneous Fibrolysis (DF) has demonstrated to improve nerve conduction studies and mechanosensitivity. The main purpose was to quantify changes in the cross-sectional area (CSA) of the median nerve, transversal carpal ligament (TCL) thickness, numbness intensity, and the subjective assessment of clinical change after DF treatment in patients with CTS. Methods: a double-blind, randomized, placebo-controlled trial was designed. A number of 44 patients (60 wrists) with CTS were randomized to the DF group or the sham group. CSA and TCL thickness variables were registered by ultrasound. Clinical variables were assessed by the visual analogue scale and GROC scale. SPSS version 24.0 for MAC was used for statistical analysis. The group by time interaction between groups was analyzed using two-way repeated measures analysis of variance. Results: The DF group reduced CSA with a mean of 0.45 mm^2^ (IC 95% 0.05 to 0.86) and TCL thickness with a mean reduction of 0.4 mm (IC 95% 0.6 to 2.1) compared to the sham group (*p* < 0.01, *p* < 0,03, respectively). Additionally, the DF group decreased the numbness intensity with a mean reduction of 3.47 (IC 95% 2.50 to 4.44, *p* < 0.01) and showed a statistically significant improvement on the GROC scale (*p* < 0.01). Conclusions: DF treatment may significantly reduce CSA and TCL thickness, numbness intensity, and improved clinical perspective. DF applied in patients with mild to moderate CTS may prevent the progression of the disease as they age.

## 1. Introduction

Carpal Tunnel Syndrome (CTS) is a median nerve compression at the wrist [1,2]. This pathology mainly affects adults of working age, and elderly patients (>65 years old) have a higher prevalence of severe CTS [3,4]. Symptoms refereed by CTS patients are pain, numbness, and sensory disturbances in the three first fingers and hand [5,6,7].

Nerve conduction studies and clinical examination are considered the gold standard for CTS diagnosis [8,9]. Recently, the validity of ultrasound (US) has been evaluated by various studies to diagnose CTS [10,11,12]. Commonly, US is used to assess the median nerve cross-sectional area (CSA) and the thickness of the transversal carpal ligament (TCL) in patients with CTS [13,14,15,16]. These measurements have shown to be correlated to nerve conduction studies, symptom severity, and functional status scores in patients with CTS [17]. An increase in the CSA of the median nerve has been correlated to worse symptoms, although cut-off values vary between studies [10,18,19].

Regarding TCL, several authors have shown hypertrophy in this ligament measured with US in patients with CTS [16,20,21]. This suggests that TCL thickening may be directly related to carpal tunnel pathomechanics [22]. In CTS patients, histological findings in adjacent connective tissue have been evidenced. Additionally, impairments in the movement of the tendons of the wrist flexor muscles near the median nerve both in the forearm and wrist have been reported [16,21,22]. Therefore, it has been suggested that the treatment of the soft tissues around the nerve could improve the symptoms and nerve conduction studies variables [23,24,25,26].

Blumenthal et al. showed that older adults with CTS present more severe nerve entrapment than younger adults [27]. Additionally, patients under 65 years have an 8.2 times increased risk of having electrophysiological impairments and it seems that the rate of axonal regeneration becomes slower with age. Thus, the treatment may not be as satisfactory in the elderly and treatment in early stages could be relevant [28]. For this reason, and as the clinical guidelines recommend conservative treatment as the best option in the initial stages of CTS, manual interventions are strategies that may be used [29,30].

Diacutaneous Fibrolyisis (DF) is a conservative instrumental technique resulting from Cyriax bases. The metallic hooks’ application allows a deeper and more specific soft tissue mobilization than manual application [31,32].

Several authors concluded that impairments in connective and soft tissues could decrease the median nerve gliding, reducing the neural function due to neural ischemia [13,33]. The mobilization of the soft tissues with the hook could improve epineural tethering in the forearm and wrist related to the median nerve, changing the compressive situation. Few studies suggested that DF could have direct mechanical effects on muscle and connective tissues [34,35,36]. Previous studies have demonstrated that DF has improved nerve conduction study test and mechanosensitivity in mild to moderate CTS patients [37,38], but it has not been demonstrated the effects on US variables, such as CSA or TCL thickness. The instrumental mobilization of the soft tissues of the forearm, wrist, and hand may decrease the median nerve compression by the neurophysiological and mechanical effects attributed in previous studies [37,38].

The primary purpose is to evaluate the effectiveness of the DF treatment in the forearm, wrist, and hand in the CSA of the median nerve. The secondary objective is to analyze the changes in the thickness of TCL measured by the US, the intensity of the numbness and the subjective assessment of clinical change after DF treatment.

## 2. Materials and Methods

### 2.1. Study Design and Ethics

A double-blind, randomized, placebo-controlled trial was designed following the CONSORT Guideline. The Clinical Research Ethics Committee of Aragon approved the study protocol with the registration number (CP13/2014) and was registered in the Clinical Trials Registry and obtained the identification number NCT04762238. It was carried out between February and June 2021 in the Miguel Servet Hospital, Zaragoza (Spain). All the participants read the written consent and signed the informed consent before the study enrollment.

### 2.2. Participants

Inclusion criteria were: diagnosis of mild or moderate CTS according to the American Academy of Physical Medicine and Rehabilitation standards by nerve conduction studies [39,40], age between 18 and 65 years, capacity to communicate their symptoms and to complete questionnaires, and sign the informed consent.

Exclusion criteria were: nerve conduction study diagnosis of severe CTS, previous surgery or pathologies in the cervical or upper limb, systemic diseases (diabetes, hypothyroidism, arthritis, obesity, renal disease, alcoholism, viral or bacterial processes), pregnancy, and oral drugs. The participants were excluded if they had received physical therapy or infiltrations in the upper extremity in the last three months. Additionally, specific contraindications for the DF treatment, such as skin disorders (for example, diaphanous and hypotrophy (or) ulcerous skin (or) dermatosis), a poor trophic state of the circulatory system or an overdeveloped network of surface veins or the consumption of antiplatelet agents.

### 2.3. Randomization and Blinding

Consecutive patients were approached for recruitment. The allocation was performed randomly using the “randomly assign subjects to treatment groups” tool of the GraphPad software (GraphPad Software, San Diego, CA, USA). The patients were divided into the DF group and the sham group (ratio 1:1). The DF group was coded with the letter “A”, and the sham group was coded with the letter “B”. An external researcher was in charge of using the software and allocate the participants in both groups.

The same physical therapist applied real and sham techniques. The blinding was maintained for examiners and patients. If the patient presented bilateral affectation, both extremities received the same intervention.

### 2.4. Interventions

Five treatment sessions were performed in both groups for all patients, each session lasted 20 min, with two sessions per week. A clinical therapist expert in the application of the DF technique performed the intervention.

**Figure 1:**
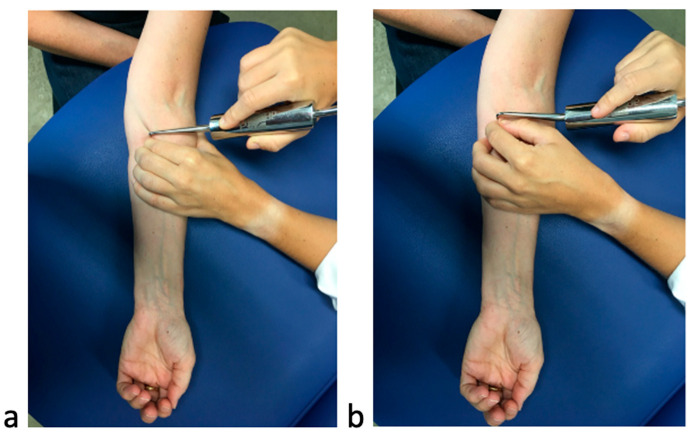
Application of independent variable ((**a**) Diacutaneous Fibrolysis: DF application on teres major muscle and (**b**) sham technique: sham application on superficial tissues).

In the DF group, the hook was applied to cover the soft tissue with the necessary pressure to move with short and fast traction in a transverse direction to the muscle fibers. The muscular septa mobilized were the forearm muscles, the pronator teres and the flexors of fingers and carpi. Firstly, the treatment was carried out in the proximal tissues of the forearm, then in the wrist, and continued to the hand’s flexor tendons and palmar fascia. [37,38] (Figure 1).

In the sham group, the application of the hook was superficial. The intervention was performed in the same regions and in the same direction. This technique has been used in previous studies and has shown adequate masking [31,32] (Figure 1).

### 2.5. Outcome Measures

Two independent examiners assessed the outcome measures. The examiners were blinded to the group allocation. An expert US examiner assessed CSA of the median nerve (main outcome variable) and the thickness of TCL. The second examiner assessed the numbness intensity and the global rating score. All the dependent variables were assessed twice, at baseline and two days after the end of the intervention, except the global rating score, which was only assessed two days after the end of the intervention. Patients were encouraged to maintain daily living and/or sports activity during the duration of the study.

#### 2.5.1. Ultrasonography Variables: CSA and TCL Thickness

In the ultrasonography assessment of CTS, a linear probe 7–12 MHz was used (ultrasound LOGICe Basic). The probe was placed perpendicular to the forearm at the trapezium-hamate level and the ridge of the trapezium corresponding to the distal level of the carpal tunnel. No additional force was applied to the probe to avoid median nerve deformation. Firstly, the US transducer was oriented perpendicularly to the palm of the participant with an axial imaging plane that provided a vision of the hook of the hamate, ridge of the trapezium, and median nerve [17,41,42].

The CSA of the median nerve was measured with the hyperechoic inner edge of epineurium reference at the pisiform and hamate bone level. The CSA was measured directly with an area measurement software, using a continuous boundary trace three times, and the mean values were quantified. This measurement procedure has shown an intraclass coefficient correlation (ICC) of 0.97 has been reported, the standard error of measurement (SEM) has been stated at 0.39 and the minimal clinically important change (MCID) at 1.73 [41].

The thicknesses of TCL on the cross-section at the level of the hamate bone were all measured. The thicknesses of TCL was calculated directly using electronic on-screen calipers just proximal to the tunnel, where the nerve was the thickest and within the carpal tunnel, where the nerve was the most flattened. The validity and accuracy of the ultrasonography measurements in CSA and thickness of TCL have been reported in previous studies [16,41,43].

#### 2.5.2. Numbness Intensity

The intensity of numbness was assessed using the visual analog scale (VAS) of 100-mm, in which 0 was considered “no numbness” and 100 was considered “maximum numbness” [44].

#### 2.5.3. Patient Global Impression Change

The patients’ clinical change was registered using the Global Rating of Change (GROC) scale. The examiners asked the patients to rate their overall change perception after the intervention. The GROC scale ranged from −7 (“a very great deal worse”) to 0 (“about the same”) to +7 (“a very great deal better”). The test–retest reliability has shown to be excellent (ICC of 0.90). The MCID has shown to be ≥+4, indicating moderate to large changes [45,46]. The GROC Scale values were grouped for statistical purposes. Values ≥+4 were defined as a significant improvement, values ≤−4 were defined as clinical worsening, and the rest of the values were defined as no change.

### 2.6. Sample Size

The sample size was calculated using the Minitab 13.0 program. The primary outcome (CSA) was chosen to perform the calculation. The standard deviation considered in previous studies was 2.4 mm, with an expected size of 1.73, power study by 80%, estimating a two-tail test, and a level of significance of 0.05 [41,47,48,49]; 30 wrists were required for each group.

### 2.7. Statistical Methods

SPSS version 24.0 for MAC was used for statistical analysis. Mean and standard deviations were calculated for quantitative variables. The normal or non-normal distribution of the variables was assessed using the Kolmogorov–Smirnov test (*p* > 0.05). Sociodemographic and clinical variables were compared between groups at baseline using a one-factor ANOVA or Mann–Whitney U test according to the normally or non-normally distributed data. The Chi-square test was used for qualitative data.

The group by time interaction between the DF group and the Sham group were analyzed using two-way repeated measures analysis of variance (ANOVA). A *p*-value <0.05 was considered statistically significant. The effect size was calculated with Cohen coefficients (d) to estimate the within-group and between group magnitudes. The Cohen value was interpreted as follows: large effect sizes, d > 0.8; moderate effect sizes, d = 0.5–0.79; and small effect sizes, d = 0.2–0.49 [50].

## 3. Results

Initially, 64 consecutive patients were recruited, and 20 patients were excluded for not fulfilling the inclusion criteria. 

**Figure 2:**
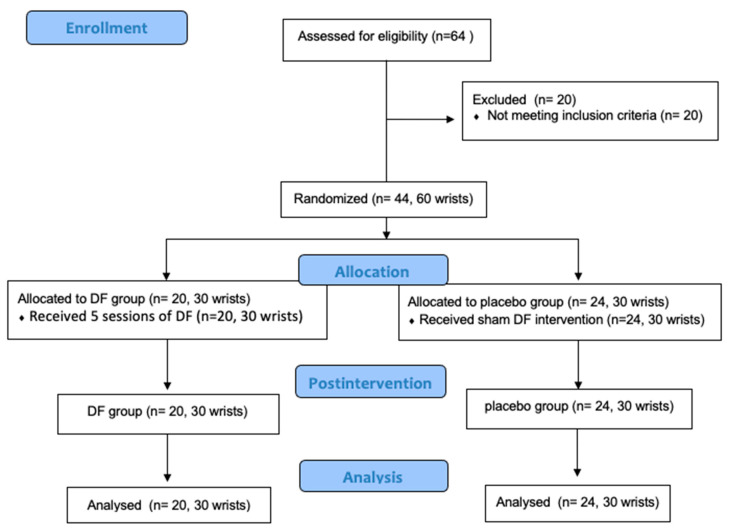
CONSORT flow diagram of the study.

Finally, 44 patients (60 wrists), 16 with bilateral and 28 with unilateral CTS, were enrolled on the trial: DF group: 20 participants (30 wrists) and sham group: 24 participants (30 wrists). The flowchart of this is shown in Figure 2. All the patients finished the study, and no adverse effects were reported.

Sociodemographic and clinical features at baseline of the patients included are shown in Table 1. No between-group differences were reported at baseline (*p* > 0.05).

Table 2 provides data between groups and within-group with statistical differences for CSA of the median nerve, thickness in TCL, and numbness intensity.

After DF treatment, significant differences between groups were found on numbness intensity (*p* ≤ 0.01; F = 25.7; Cohen’s d = 1.5), CSA of median nerve (*p* ≤ 0.01; F = 6.7; Cohen’s d = 0.8), and thickness TCL (*p* ≤ 0.03; F = 6.4; Cohen’s d = 1.1).

**Figure 3:**
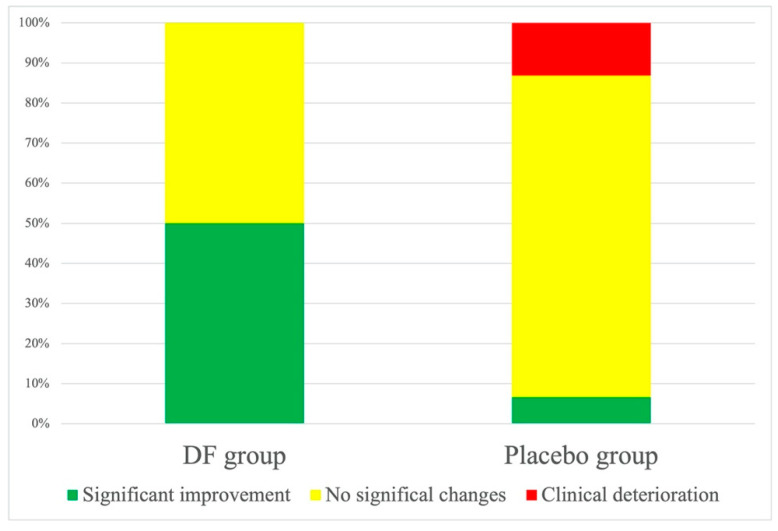
Graphical representation of subjective clinical change using the GROC scale.

Concerning the GROC scale, none of the patients in the DF group reported clinical deterioration and 50% reported subjective improvement (n = 15), while 50% reported no change (n = 15). In the sham group, 13.2% reported clinical deterioration (n = 6), 80.2% no change (n = 21), and 6.6% reported subjective improvement (n = 3) (Figure 3). Differences between the DF and sham groups were statistically significant (*p* < 0.01).

## 4. Discussion

This study aimed to investigate the effects of soft tissue mobilization applying DF on US measurement, numbness intensity, and the global rating of change in patients with CTS. Results showed that the treatment based on soft tissue mobilization with five sessions of DF reduced the CSA of the median nerve, the TCL thickening and numbness intensity, showing a significant improvement in the GROC score.

The patients included in this study demonstrated similar characteristics at baseline to previous studies [17,23,37,38]. The mean of the numbness intensity showed values that reflect the clinical findings in patients with CTS [7]. The CSA values reported at baseline were higher than the cut-off point described for CTS diagnosis [10,12].

The present study has shown a statistically significant decrease in the CSA after DF treatment. The reduction of the CSA of the median nerve found in this study was higher than the standard error of measurement (0.39) [41].

The results achieved in this study are in accordance with other clinical trials that demonstrated the use of the US to control the improvements after therapies. However, there is a lack of agreement on the correlation between CSA and clinical and electrophysiological scales [48].

Previous studies tried to modify the structures around the nerve in the carpal tunnel. Bueno-Gracia et al. showed morphological changes in the carpal tunnel and the CSA after mobilizing the carpal bones [51,52,53]. Other studies aimed to analyze the effects of conservative treatments, such as taping, splint, or ultrasounds, did not demonstrate significant improvements in CSA of the median nerve [42,49]. Previous studies reported improvements after a surgical intervention or corticosteroid infiltration on CSA assessment [54,55]. It is important to consider the possible adverse effects derived from these interventions. In our study, the mean median nerve CSA after treatment was 8.8 mm^2^, which revealed a significant decrease between groups, treating only soft tissues related to carpal tunnel. 

There is no evidence of the effects on TCL after conservative treatment, and for this reason, there is no possibility to compare the results. However, the clinical changes achieved after DF intervention may support evidence of the effects on the structures directly related to the carpal tunnel. This fact could be related to the hypothesis that nearby tissues could have an important role in carpal tunnel compression.

DF has shown to effectively decrease symptoms and improve function in patients with different musculoskeletal disorders. The goal of this technique is to support movement and facilitate mobilization. It has been proposed that the soft and connective tissues and epineural tethering in the forearm and wrist could reduce the median nerve gliding. This fact may condition in an increment of pressure on the nerve causing neural ischemia [33], local metabolic disturbances, and disorders of neural function [56]. The increase of the movement between the muscles by the mobilization of the intermuscular with the hook could decrease the pressure developing in this area. In this way, it helps blood circulation, decreasing the adhesion and tenderness in the damaged area.

The presence of numbness involving the first four fingers and hand is the most characterized symptom of patients with CTS [57,58]. Moreover, previous studies have demonstrated that the distribution of numbness could reflect the degree of nerve damage [59]. 

The outcomes achieved on symptoms are similar to other studies that applied non-surgical interventions based on soft tissue treatment in patients with CTS [23,24,60,61,62]. 

In our study, the DF group decreased the mean numbness intensity from 3.7 to 0.23 (0–10), showing a reduction higher than 30% determined as a clinically relevant change [44].

This study has demonstrated that DF intervention achieved significant subjective clinical change compared to sham. The natural evolution of CTS in untreated patients, as the placebo group shows in this study, is no change or deterioration in their symptoms and other clinical assessment [63,64,65].

From a clinical point of view, the results achieved in this clinical trial found that the symptoms and signs of patients with CTS are related to soft tissue dysfunctions in the forearm, wrist, and hand. The intervention based on five sessions of DF showed statistical and clinical benefits in CSA, numbness intensity, and clinical perspective. The US measurements seem to be a promising outcome variables to control the progression of the pathology in clinical practice. According to these results, the treatment of the forearm, wrist, and hand soft tissues should be considered in patients with mild to moderate CTS affectation and may delay or even prevent progression to severe cases in elderly people.

Several limitations should be considered. First, only patients under 65 years old diagnosed with mild to moderate CTS were included, so the results cannot be extrapolated to severe affectations. Second, no medium- or long-term evaluations were considered. Additionally, the effects showed were following the DF technique. However, CTS management should be multimodal. Finally, both wrists of the patients diagnosed with bilateral CTS were included in the same group to guarantee the correct blinding, so this could be a limitation in the randomization process.

Future studies should investigate the long-term effects of DF in severe cases and perform prospective studies assessing the possible prevention of the progression. Additionally, they may evaluate the effects of different treatment combinations in different CTS degrees of affectation.

## 5. Conclusions

It seems that the conservative treatment consisting of DF application in soft and connective tissues of the hand, wrist, and forearm could decrease the CSA of the median nerve in the carpal tunnel. Additionally, DF may reduce the TCL thickness and the intensity of symptoms. DF applied in patients with mild to moderate CTS prevent the disease’s progression in the elderly. The results should be taken with caution due to the limitations of the study.

## Figures and Tables

**Table 1:** Between-groups comparisons at baseline (n = 44 patients, 60 wrists).

Features	DF Group n = 20 (30 Wrists)	Sham Group n = 24 (30 Wrists)	Significance
Age	44.17 (10.00)	48.9 (8.69)	*p* = 0.063 ^a^
Sex (female/male)	24/6	26/4	*p* = 0.731 ^b^
Duration of suffering (months)	24.43 (22.78)	27.77 (37.73)	*p* = 0.346 ^c^
Body Mass Index (BMI) (Kg/m^2^)	24.76 (2.8)	25.84 (2.92)	*p* = 0.203 ^a^
Work activity (outside home) Active/Not working	28/2	27/3	*p* = 0.891 ^b^
Use wrist in sports activity Yes/No	8/22	5/25	*p* = 0.298 ^b^
Wrist circumference (cm) (mean and SD)	16.00 (1.25)	16.05 (1.02)	*p* = 0.731 ^a^

DF group: Diacutaneous Fibrolysis group; BMI: body mass index; ^a^ Mann–Whitney U test; ^b^ Chi-square test; ^c^ One-factor ANOVA.

**Table 2:** Mean values before and after intervention, mean changes and magnitude of numbness, CSA, and thickness of TCL (n = 44 patients, 60 wrists).

Outcomes	Baseline	End of Treatment	Within-Group Changes (95%IC)	Within-Group Effect Sizes	Between-Group *p*-Values	Between-Group Effect Sizes
**VAS Numbness (0–10)**						
DF group	3.7 (2.64)	0.23 (0.54)	3.47 (2.50–4.44)	1.8	<0.01	1.46
Sham group	2.95 (2.2)	2.87 (2.5)	0.08 (−0.97–1.1)	0.0		
**CSA (mm^2^)**						
DF group	9.3 (1.4)	8.8 (1.5)	0.45 (0.05–0.86)	0.34	<0.01	0.70
Sham group	9.7 (1.0)	9.7 (1.0)	−0.02 (−0.22–0.18)	−0.2		
**Thickness TCL (mm)**						
DF group	22.0 (0.02)	20.0 (1.6)	0.4 (0.6–2.1)	1.0	<0.03	0.89
Sham group	21.3 (0.01)	21.3 (1.3)	−0.28 (−0.57–0.02)	−0.2		

DF: Diacutaneous Fibrolysis; VAS: Visual analog scale; CSA: cross-sectional area; TCL: transversal carpal ligament.

## Data Availability

The anonymized data are available from the author upon reasonable request.
